# Our Transactions

**Published:** 1885-12

**Authors:** 


### OUR "TRANSACTIONS."

THE following handsome compliment is only one of many that have been bestowed upon the copy of Transactions of our State Association for 1885:

"The Medical Association of Texas, though among the junior State Medical organization of America, shows a commendable degree of enterprise, progress and vigor—far in advance of the Associations and State Societies of many other States—whose age alone should have kept them well in advance. We heartily congratulate our professional brethren in this great Empire State of the West on so handsome and in every way commendable a volume of Transactions as the outcome of their last annual meeting." * * * * * *

"The typographical execution of the work, the handsome cloth binding, and good paper, make it suitable to the most pretentious library."—*Southern Practitioner.*

